# What lies beneath? Molecular evolution during the radiation of caecilian amphibians

**DOI:** 10.1186/s12864-019-5694-1

**Published:** 2019-05-09

**Authors:** María Torres-Sánchez, David J. Gower, David Alvarez-Ponce, Christopher J. Creevey, Mark Wilkinson, Diego San Mauro

**Affiliations:** 1 Department of Biodiversity, Ecology and Evolution, Complutense University of Madrid, 28040 Madrid, Spain; 2 Present address: Department of Neuroscience, Spinal Cord and Brain Injury Research Center & Ambystoma Genetic Stock Center, University of Kentucky, Lexington, KY 40536 USA; 3 Department of Life Sciences, The Natural History Museum, London, SW7 5BD UK; 4 Department of Biology, University of Nevada, Reno, NV 89557 USA; 5 Institute for Global Food Security, Queen’s University Belfast, University Road, Belfast, BT7 1NN Northern Ireland, UK

**Keywords:** Ecological opportunity, Gene ontology, Gymnophiona, Positive selection signatures, Vertebrate evolution

## Abstract

**Background:**

Evolution leaves an imprint in species through genetic change. At the molecular level, evolutionary changes can be explored by studying ratios of nucleotide substitutions. The interplay among molecular evolution, derived phenotypes, and ecological ranges can provide insights into adaptive radiations. Caecilians (order Gymnophiona), probably the least known of the major lineages of vertebrates, are limbless tropical amphibians, with adults of most species burrowing in soils (fossoriality). This enigmatic order of amphibians are very distinct phenotypically from other extant amphibians and likely from the ancestor of Lissamphibia, but little to nothing is known about the molecular changes underpinning their radiation. We hypothesised that colonization of various depths of tropical soils and of freshwater habitats presented new ecological opportunities to caecilians.

**Results:**

A total of 8540 candidate groups of orthologous genes from transcriptomic data of five species of caecilian amphibians and the genome of the frog *Xenopus tropicalis* were analysed in order to investigate the genetic machinery behind caecilian diversification*.* We found a total of 168 protein-coding genes with signatures of positive selection at different evolutionary times during the radiation of caecilians. The majority of these genes were related to functional elements of the cell membrane and extracellular matrix with expression in several different tissues. The first colonization of the tropical soils was connected to the largest number of protein-coding genes under positive selection in our analysis. From the results of our study, we highlighted molecular changes in genes involved in perception, reduction-oxidation processes, and aging that likely were involved in the adaptation to different soil strata.

**Conclusions:**

The genes inferred to have been under positive selection provide valuable insights into caecilian evolution, potentially underpin adaptations of caecilians to their extreme environments, and contribute to a better understanding of fossorial adaptations and molecular evolution in vertebrates.

**Electronic supplementary material:**

The online version of this article (10.1186/s12864-019-5694-1) contains supplementary material, which is available to authorized users.

## Background

Understanding the diversity of life and how species have evolved into their different and specialized forms is an ultimate goal of evolutionary biology. The events that lead to macroevolutionary diversification by adaptive radiation have been related to ecological opportunities: the availability of new ecological resources for exploitation [1–3]. The potential for ecological opportunities to trigger adaptive radiation (diversification of species from a common ancestor into different ecomorphological forms) is widely recognised (e.g. [4–8]). Phenotypic evolutionary changes accumulated during adaptive radiations ultimately have a molecular basis that can involve a variety of genetic changes, including gene gain and loss, beneficial mutations, regulatory changes or other innovations [9–11]. As more genomic data becomes available, a better understanding of the evolutionary mechanisms underpinning biodiversity should follow. Molecular evolutionary processes can be investigated by studying regulatory and/or functional elements of genomes. In protein-coding genes, sources of evolutionary variation can be explored by comparing rates of nucleotide substitutions at synonymous (dS) and non-synonymous (dN) sites; substitutions in those latter sites result in a change of amino acid sequence and consequently can result in change of phenotype. The ratio between these rates, ω (ω = dN/dS), provides a widely used means of identifying selective pressures in proteins [12].

Adaptive radiation of vertebrates is in part explained by genetic changes that allowed new functions to emerge [13–15], increasing the fitness of the organisms in new environments. One of these environments, the soil, presents several restrictive conditions, including low levels of light, high resistance to locomotion, low airborne transmission of sound and scent, and low oxygen (O_2_) and high carbon dioxide (CO_2_) levels (hypoxia and hypercapnia respectively). In addition, many microorganisms (fungi, protozoans, bacteria) and diverse invertebrates (often pathogenic) abound in especially humid and thermally stable soils [16]. Despite these challenges, several groups of vertebrates are well adapted to life in soil [17–19], including one of the most ancient lineages of extant terrestrial vertebrates, the caecilian amphibians that radiated in the edaphic environment during the early Mesozoic [20, 21]. Caecilians (order Gymnophiona) are limbless, elongate, mostly tropical amphibians. Adults of most species burrow in soil. Many other extant amphibians spend time in soil but feed and breed above ground [22, 23]. In contrast, many adult terrestrial caecilians are highly fossorial, dedicated burrowers that feed and breed within moist soils [24]. Terrestrial caecilians inhabit different layers of soil, from leaf litter to deeper strata, while species of one family (Typhlonectidae) are secondarily semi- or fully aquatic [22]. Caecilian evolution has clearly involved the colonisation of tropical soils. We hypothesise that, as well as providing distinctive challenges, the soil offered new ecological opportunities to caecilians with new resources and absence of, or reduction in, competitors and predators, perhaps similar to emergent islands [25–28], newly formed lakes [29, 30], and post-mass extinction environments [31, 32] for other organisms. Regions with high above-ground biodiversity, such as the tropics, exhibit low below-ground biodiversity [33] where caecilians might have encountered lower competitive pressure. In addition of the suggestive reduced competition, soil is potentially more stable and less subject to harmful fluctuations in humidity and temperature. Ancestral caecilians adapted to life in soil, developed important innovations and diversified. Given that fossoriality is a derived condition among amphibians, several morphological features of caecilians are clearly adaptations to life in soil, some of which are shared convergently with other edaphic animals. These include modified skull architecture for head-first burrowing and feeding underground [34], elongated limbless bodies with modified axial musculature [35, 36], reduced visual and hearing systems, and novel sensory tentacles [37–39]. The molecular changes underlying the evolutionary origin and diversification of caecilians remain unexplored thus far. In this study, we investigated molecular processes involved in the exploitation of (i) soil surface habitats, (ii) deeper soil habitats, and (iii) freshwaters and associated muds.

**Fig. 1:**
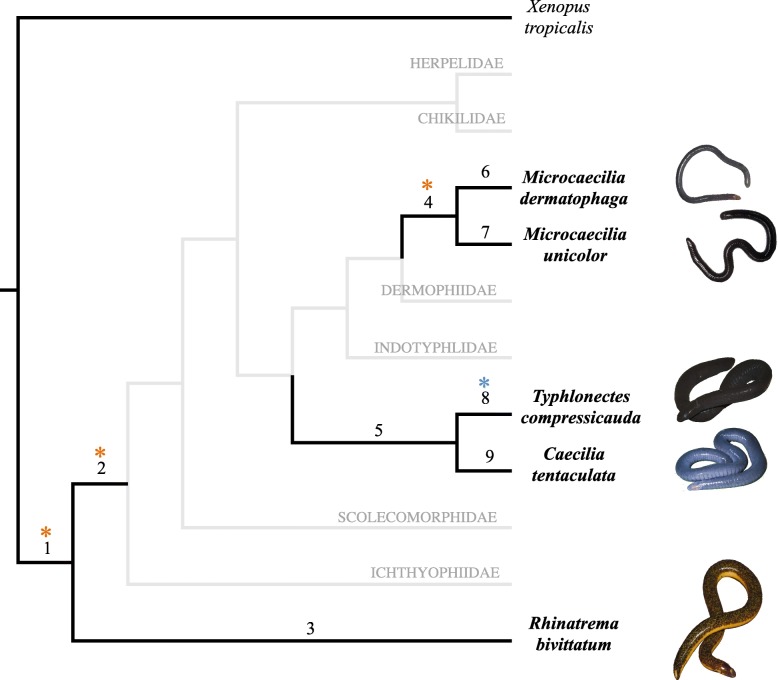
Phylogenetic tree used in the tests of positive selection. Branches used as foreground branches in the different tests are indicated with numbers as follows: 1: Gymnophiona branch, 2: Teresomata branch, 3: *R. bivittatum* branch, 4: *Microcaecilia* branch, 5: *Caecilia + Typhlonectes* branch, 6: *M. dermatophaga* branch, 7: *M. unicolor* branch, 8: *T. compressicauda* branch and 9: *C. tentaculata* branch. Hyphothesied ecological opportunities are marked with asterisks. Phylogeny based on [40] and [69]. Note that the sampling includes species from both sides of the basal divergence within Gymnophiona, so that branch 1 terminates in the last common ancestor of all extant caecilians. (Pictures credit: MW)

Recently, reference transcriptomes for several species of caecilians have been generated [21, 40], providing an opportunity to explore genomic changes in caecilian amphibians. Here, we analyse the protein-coding sequences from transcriptome data for nine different tissue types (foregut, heart, kidney, liver, lung, muscle, skin, spleen, and testis) of five species of Neotropical caecilians [40] that occur in a range of habitats (DJG, MW, DSM pers. obs.). The semi-fossorial species *Rhinatrema bivittatum* (Guérrin-Méneville, 1838) is encountered mostly in more superficial layers of soils as well as on the surface after heavy rain. *Caecilia tentaculata* Linnaeus, 1758 appears to be a much stronger burrower based on its more heavily ossified skull [34], but it is also encountered on the surface after heavy rains. *Typhlonectes compressicauda* (Duméril & Bibron, 1841) is a fully aquatic species that can burrow in soft substrates. *Microcaecilia unicolor* (Duméril, 1861) and *M. dermatophaga* Wilkinson, Sherratt, Starace & Gower, 2013 are more dedicated burrowers not seen on the surface and mostly found in deeper layers of the soil. The sampled caecilians include species from both sides of the basal divergence within Gymnophiona belonging to four of the ten currently described families [41, 42] of the order (Rhinatremidae, Typhlonectidae, Siphonopidae and Caeciliidae), and their phylogenetic history encompasses several major shifts in caecilian evolution. We have compared nucleotide substitution rates of candidate groups of orthologous protein-coding genes for these five caecilian species in order to identify genes that potentially have, at some time, been under positive selection. The sampled caecilians allow us to explore nine different branches of the caecilian tree of life (Fig. 1) covering the evolutionary periods in which caecilians first adapted to life in soil, and subsequently adapted to deeper soils and to aquatic environments. We identified signatures of positive selection in several protein-coding genes on all branches. Some of these candidate genes could be involved in the adaptive radiation of caecilian amphibians, plausibly in the adaptation to fossoriality, and in the evolution of their special innovative traits.

## Results

We identified 8540 candidate groups of one-to-one orthologous protein-coding sequences (ranging in size from 138 to 94,440 bp) among the sampled caecilian species (*R. bivittatum*, *C. tentaculata*, *T. compressicauda, M. unicolor* and *M. dermatophaga*) and the frog outgroup (*Xenopus tropicalis* Gray, 1864). Through branch-site model comparisons, we detected 168 genes with signals of potential adaptive molecular evolution along the nine sampled branches (Fig. 1) of the caecilian evolutionary tree. From the identified sites (the fraction of codons with ω > 1) in those 168 genes, we found an overall 4.39% of the codons under positive selection at contiguous positions, which were mainly located in genes with a large number of codons involved in the signature of selection. All the alignments of the 168 genes with signatures of positive selection presented a GUIDANCE2 alignment score higher than 0.96 with the exception of one alignment with a value of 0.924565 (ENSXETG00000018913; see Additional file 1: Table S1 column GUIDANCE2 alignment score). The alignment confidence scale of the GUIDANCE2 showed a high confidence in the great majority of the analysed sites of our alignments (95.45% of the sites present the maximum alignment confidence value). We characterized the genes with evidence of sites under positive selection using the functional annotation of their homologous genes in *X. tropicalis*. These annotations are summarized numerically in Table 1 (see Additional file 1: Table S1 for more details).

**Table 1:** Number of genes under positive selection

Foreground branch	Branch number	Genes under positive selection (FDR < 10%)	Genes with description	Genes with GO	Biological process domains	Molecular function domains	Cellular component domains
Gymnophiona	1	50	47	43	96	84	75
Teresomata	2	8	8	7	13	16	16
*Rhinatrema bivittatum*	3	17	17	15	31	29	22
*Microcaecilia*	4	13	12	11	34	33	19
*Caecilia* + *Typhlonectes*	5	15	14	15	28	35	19
*Microcaecilia dermatophaga*	6	33	30	31	74	72	44
*Microcaecilia unicolor*	7	16	15	15	48	56	28
*Typhlonectes compressicauda*	8	18	17	16	34	32	27
*Caecilia tentaculata*	9	7	7	7	23	15	16

**Fig. 2:**
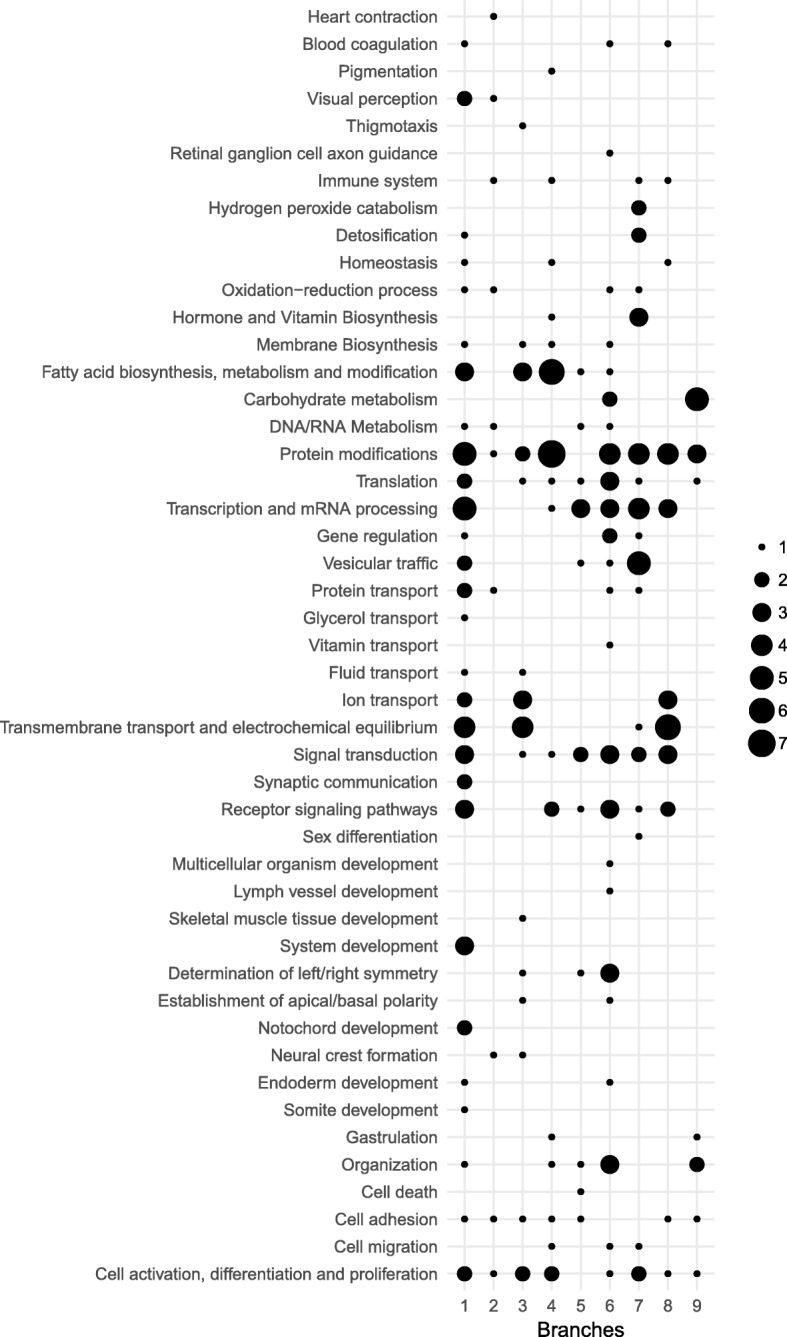
General categories of biological processes from gene ontologies (GO) related to the genes under positive selection. For each of the sampled branches, the relative number of different annotated GO terms (a proxy of the number of identified genes under positive selection) under a general biological processes is symbolized by the different circle sizes (see legend)

**Fig. 3:**
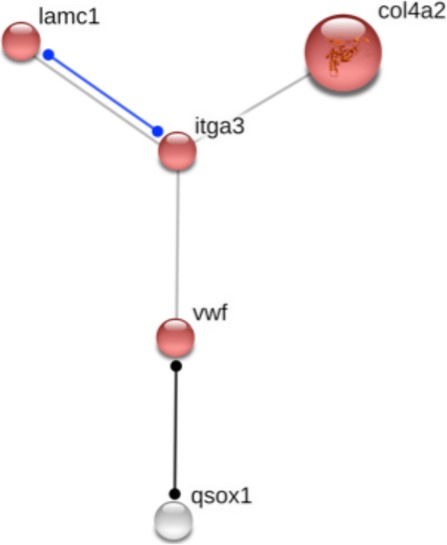
Protein-protein interaction (PPi) network predicted from the positive selected genes of the Gymnophiona branch (branch 1) that are involved in the ECM-receptor interaction pathway with a binding interaction (blue line) between lamc1 and itga3, and a reaction interaction (black line) between vwf and qsox1 (the latter protein-coding gene is part of a second shell of interactions)

The vast majority of the genes inferred to have been positively selected (153 genes) were associated with gene ontology (GO) terms that involve: 247 out of 4301 biological processes annotated in *X. tropicalis*, 74 out of 856 cellular components, and 170 out of 2041 molecular functions (Table 1 and Additional file 1: Table S1). A total of nine of those genes with GO information were classified as novel or uncharacterised without gene description in the Ensembl database. For these nine genes (*sult1c1*, *col16a1*, *slc18a1*, *ano9*, *fr47*, *plg*, *tmprss2*, *pacsin1*, and *inmt*; see Additional file 1: Table S1) further information was retrieved from the Non-redundant protein sequences database. GO information for biological process domains of the positive selected genes was gathered for the nine sampled branches of the caecilian evolutionary tree. A total of 47 general categories were created from the summarized GO terms of the REVIGO exploratory networks using semantic connectivity of the GO terms [43]. In Fig. 2 the relative number of different GO terms is used as a proxy for the number of genes under a general biological processes; these are symbolized to show the different processes for which positive selected genes are involved for each branch. Despite the semantic connectivity, no functional enrichment and protein-protein interactions (PPIs) were found within any of the sets of genes under positive selection on each branch, and only one association network on the Gymnophiona branch presented evidence (although not statistically significant) of functional enrichment linked to four genes considered to be involved in extracellular matrix (ECM) interactions (Fig. 3). GO terms of cellular component domains, which reflect subcellular location, related to cellular membrane and ECM elements (membrane [GO:0016020], integral component of membrane [GO:0016021], ECM [GO:0031012], extracellular region [GO:0005576], extracellular space [GO:0005615] and proteinaceous ECM [GO:0005578]) were the most common terms assigned to the positively selected genes. Many of these genes are also associated with extracellular processes, transmembrane signaling and transport terms of the biological process GO domain (see biological process categories: Receptor signaling, Synaptic communication, Signal transduction, Transmembrane transport and electrochemical equilibrium, and Vesicular traffic in Fig. 2; and find further information in the Additional file 1: Table S1). Finally, functional characterization of the genes under positive selection was completed with caecilian tissue expression information available for nine different tissue types (for detailed information see Additional file 1: Table S1 Tissue expression column). Only five of the genes with signatures of positive selection showed tissue specificity expression (*col17a1* with more than 95% of its Transcripts Per Million (TPM) in the skin; *tmem27* in the kidney; and *f2*, *klkb1* and *plg* in the liver).

Just two of the nine studied branches of the phylogeny account for almost 50% of the identified signatures of positive selection: the branch subtending the clade comprising all sampled caecilians, hereafter the “Gymnophiona branch” (branch 1 in Figs. 1, 50 genes, 29.58%: *acot2, wdr1, slc34a2, sod3, col4a2, akr1a1, als2cl, nup155, c10orf35, ddx17, adamts7, nckipsd, esyt1, msn, aqp9, slc22a31, rph3a, lamc1, tet2, gstcd, nup153, gdpd5, tacc2, klhdc10, golga1, pigr, gigyf1, cul9, cdhr2, hprt1, cgn, itga3, p2ry11, ptprh, SPEN, qsox1, vwf, cdk12, tbrg4, tcf19, spg11, rps13, gsto2, tnrc6a, cp, col17a1*, *acadvl*, *sult1c1*, *col16a1* and *slc18a1*; see Additional file 1: Table S1), and the terminal branch subtending *M. dermatophaga* (branch 6 in Figs. 1, 33 genes, 19.52%). There are significantly more genes with a signal of positive selection on the terminal branches subtending *M. dermatophaga* and *T. compressicauda* than on the terminal branches subtending their respective sister species in the sampled phylogeny (*Microcaecila* sister group: branches 6 and 7, *Typhlonectes*-*Caecilia* sister group: branches 8 and 9, with two-tailed binomial tests *p*-values 0.021 and 0.043 respectively). Different proportions of genes under positive selection were found also associated with branches (branches 1, 2, 4 and 8 in Fig. 1) that represent hypothesized ecological opportunities. Some of these positive selected genes in the four above-mentioned branches could have been involved in the adaptation to the new edaphic environments that caecilians were colonising. In addition to the 50 genes on the Gymnophiona branch, that is related to the initial hypothesized ecological opportunity in soil surface habitats, we found eight genes (*fam3b, aoc3, mbd5, hgs, masp1, pcdh7, tnc*, and *sypl1*; see Additional file 1: Table S1) with signatures of positive selection (5.32% of the total of genes with signatures of positive selection in this study) on the branch in which the hypothesized conquest of deeper soil habitats might have happened (branch 2, Fig. 1 hereafter “Teresomata branch”). The adaptation to freshwater habitats and associated muds that occurred on the branch subtending *T. compressicauda* (branch 8 in Fig. 1) could have been mediated by some of the 18 genes under positive selection on this branch (*f2, col4a1, slc30a10, camkmt, klkb1, mios, polr2a, prkag3, cwc22, ate1, myh4, thoc5, arhgap33, clcn3, fam13a, adgrg6, dsg2*, and *fr47*; see Additional file 1: Table S1). Finally, adaptation to more extreme fossoriality linked to the branch subtending *Microcaecilia* (branch 4 in Fig. 1, hereafter “*Microcaecilia* branch”) could have been facilitated by some of the 13 genes (*pinx1, col4a2, fam3b, iqsec2, ddx24, mrps7, elovl5, ca5b, yes1, basp1, tspan36, acp1,* and *plg*; see Additional file 1: Table S1) with signatures of positive selection (7.73% of the total of genes with signatures of positive selection in this study) on this *Microcaecilia* branch.

Finally, several of the positively selected protein-coding genes might be related (potentially causally) to unique traits of caecilian amphibians beyond their adaptations to the four hypothesised new environments. Among them, six protein-coding genes annotated as collagen chains were found to bear evidence of positive selection on several branches (*col4a2* on the Gymnophiona and the *Microcaecilia* branches; *col17a1* and *col16a1* on the Gymnophiona branch; *col4a1* on the *T. compressicauda* branch; *col12a1* on the *M. dermatophaga* and the *M. unicolor* branches; and *col5a2* on the *R. bivittatum* and the *M. unicolor* branches; see Additional file 1: Table S1); nine genes related to lipid metabolism and fatty acid metabolism (*acot2* on the Gymnophiona branch; *gdpd5* on the Gymnophiona branch; *plpp1* on the *R. bivittatum* branch; *elovl5* on the *Microcaeclia* branch; *sptlc3* on the *M. unicolor* branch; *cyp17a1* on the *M. unicolor* branch; *lcat*, *asah1*, and *cers6* on the *M. dermatophaga* branch; see Additional file 1: Table S1, and biological process category: Fatty acid biosynthesis, metabolism and modification in Fig. 2); and at least five components involved in immune system and related mechanisms (*tet2* on the Gymnophiona branch; *masp1* on the Teresomata branch; *enpp3* on the *Caecilia* + *Typhlonectes* branch; *yes1* on the *Microcaecilia* branch; *fyn* on the *M. unicolor* branch; see Additional file 1: Table S1, and biological process category: Immune system and Homeostasis in Fig. 2).

## Discussion

### General view of caecilian molecular evolution

Our analyses identified 168 protein-coding genes with signatures of having been under positive selection at least once during the evolution of caecilians. The reliability of the selection signals is supported by the adequacy of the alignments, quantified by the GUIDANCE2 alignment score [44], and the small proportion of adjacent codons with ω > 1, which endorse the independence of the nucleotide changes that is required by the applied selection tests [12]. The identified genes represent only 1.97% of the total surveyed genes, a small proportion compared with studies of other taxa [45–47], and presented a lack of connectivity that perhaps reflects lack of knowledge about the identified genes [43]. These 168 candidate genes under positive selection are almost certainly a substantial underestimate due to our conservative selection of orthologous sequences (only those present in every species, including *X. tropicalis*; no paralogs within species; and stringent filtering; see Methods section) intended to reduce false positives caused by alignment artefacts, to which positive selection inference methods are known to be sensitive [48]. We also are probably missing signal from genes whose evolution history is not congruent with the species tree topology [49]. An additional source of underestimation in the detection of positive selection could come from genes that are saturated [50].

Valuable insights into the molecular evolution of caecilians can be extracted from the functional annotations of the genes bearing signatures of positive selection. The high prevalence of GO terms related to cell membrane and its integral components in the set of genes with signatures of positive selection seems to underline the important role of the membrane components during the evolution of caecilian amphibians, and is consistent with positive selection signals found in other species of vertebrates [51, 52] and with previously identified regulatory innovations related to extracellular signaling in the evolution of other major tetrapod groups [53]. Molecular changes in functional elements of the cell membrane and the ECM are likely an additional important genetic aspect of vertebrate macroevolution.

### Ancient genetic toolkit for caecilians

The evolutionary changes on the Gymnophiona branch occurred subsequent to the divergence of caecilians from the other extant amphibians, leading to the last common ancestor of all extant caecilians. During this period in evolution, caecilian ancestors would have started to colonise soil environments and exploit the ecological opportunities they provided, and we would expect molecular changes linked to fossorial adaptation. From the 50 genes with signatures of positive selection that are involved in 96 biological processes based on their GO annotation grouped in 28 general categories (Table 1, Fig. 2 and Additional file 1: Table S1), we highlight the identified genes involved in development-related processes (*lamc1, tet2, nup153, tacc2*, *spg11*, see Additional file 1: Table S1 and Fig. 2); and in oxidation-reduction (redox) processes (*sod3, akr1a1, qsox1, cp*, see Additional file 1: Table S1 and Fig. 2).

Related to development, one of the candidate genes deserves special mention, a component of the extracellular glycoprotein matrix of the membrane, the laminin subunit gamma 1 (*lamc1*), which is essential for basement membrane assembly during mice embryogenesis [54–56]. The *lamc1* gene is associated with several development and morphogenesis processes (GO:0007420, GO:0048854, GO:0048731, and GO:0061053). Additionally, *lamc1* is one of the four elements of the detected functional gene-network (Fig. 3). Its function is linked to ECM interaction mechanisms [57], such as cell adhesion and cell-to-cell communication (ECM-receptor interaction: KEEG pathway ID 04512; see Fig. 3). Among other functions, *lamc1* is related to light perception (GO:0050908), retinal and eye development (GO:0031290, GO:0001654, respectively), and optokinetic behavior (GO:0007634). The gene *lamc1* has also been related to mechanosensitive processes in zebrafish [58] and studied as part of a set of important genes for perception in mammals [59]. Unlike other extant amphibians, caecilians are rod-only monochromats with small eyes covered by skin and sometimes also bone [60]. Light is not only important for visual perception, but also plays other important roles controlling, for example, the circadian rhythms vital for synchronization of biological cycles [61]. We hypothesize that molecular innovation in *lamc1* might be involved in sensorial adaptation, perhaps related to circadian rhythms, in underground environments.

Oxidation-reduction (redox) processes are associated (by GO terms) with four protein-coding genes inferred to be under positive selection on the Gymnophiona branch. Environmental conditions could have driven the emergence of molecular changes to tolerate chronic low O_2_ and high CO_2_ levels that characterise soils [16]. At higher concentrations, CO_2_ is converted to acid by ionic dissociation and can cause oxidative stress, in turn related to disease and ageing [62]. Additionally, O_2_ deprivation can affect synaptic transmission and ultimately cause cell death by cytosolic accumulation of calcium ions (Ca^2+^; [63]). The gene *rph3a* (see Additional file 1: Table S1) is a candidate gene under positive selection that could be related to redox processes. It is involved in the regulation of synaptic vesicle traffic that mediates the release of a neurotransmitter when Ca^2+^ cytosolic levels rise (GO:0048854: calcium ion-regulated exocytosis of neurotransmitter). Redox processes innovations might have contributed to the development of better protective mechanisms to increased cytotoxic threats in the edaphic atmosphere. Similar adaptations have been reported in the most studied fossorial mammal: the naked mole-rat *Heterocephalus glaber* Rüppell, 1842, where hypoxia experiments have revealed an attenuation of the accumulation of intracellular calcium [64] and the importance of redox processes [65, 66] during O_2_ deprivation. Naked mole rats have a surprisingly low metabolic rate in comparison with other mammals. Caecilians also maintain a low metabolism, notably lowest among extant amphibian groups [67, 68].

### Evolvability in Teresomata ancestors

After the colonization of surface soil habitats and initial diversification of caecilians, the origin of the Teresomata probably involved colonisation of and adaptation to deeper soil habitats and a second wave of ecological opportunity. Several major events in caecilian evolution occurred along the Teresomata branch (branch 2 in Fig. 1), including the loss of a free-living larval stage and the origin of maternal feeding [69]. Given that number of evolutionary changes, surprisingly only eight genes were found with signatures of positive selection. Some of these genes were associated with different GO terms including redox processes (see Additional file 1: Table S1 and Fig. 2). Given that gas exchange (O_2_ and CO_2_) becomes increasingly hampered deeper within soils, redox processes innovations, in this case by changes in the *aoc3* gene (candidate gene under positive selection in the Teresomata branch), might have helped caecilians to cope with the more and more extreme conditions in this habitat. The highlighted gene (*aoc3*) encodes vascular adhesion protein 1, whose expression increases under hypoxia [70].

Within Teresomata, a major ecological shift occurred in the sampled evolutionary tree along the terminal branch subtending *T. compressicauda* (branch 8 in Fig. 1), with the evolution of fully aquatic adults and viviparity*.* Among the genes identified on the *T. compressicauda*, the gene *fam13a* is involved in signal transduction (GO:0007165) and has been related to different lung diseases [71, 72] with its activity induced by low levels of O_2_ [73]. While cutaneous gas exchange is important in Amphibia [74], *T. compressicauda* has the largest lungs of any caecilian [75], is reported to have more than 90% pulmonary oxygen uptake [76], and is able to tolerate hypoxic and hypercapnic conditions [77]. Thus, changes in *fam13a* might be related to enhanced pulmonary function.

### The expert burrowers

The genus *Microcaecilia* Taylor, 1968 with 14 described species so far (two sampled in our study, *M. unicolor* and *M. dermatophaga*) is the most speciose genus of caecilians after *Ichthyophis* Fitzinger, 1826 (50 species) and *Caecilia* Linnaeus, 1758 (34 species). *Microcaecilia* have bullet shaped heads and heavily ossified skulls with prominent snouts and rudimentary eyes that are covered by bone [78]. They are the most dedicated burrowers among our sampled taxa. This more extreme fossoriality perhaps led to another wave of ecological opportunity for caecilian radiation. Among the genes found under positive selection on the *Microcaecilia* branch, the gene *pinx1* inhibits telomere elongation (GO:0010521: telomerase inhibitor activity; GO:0051974: negative regulation of telomerase activity; GO:0003676: nucleic acid binding; [79]) and has been related to aging, also found to have been under positive selection in a study of molecular adaptations to fossorial life in African mole-rats [47]. Changes in *pinx1* might be an indication of a relatively extended lifespan in *Microcaecilia* compared to other amphibians, as in mole-rats among mammals. However, little is known about longevity in caecilians, especially in the wild [80].

Another gene inferred to have been under positive selection along the *Microcaecilia* branch that drew our attention is linked to pigmentation by the GO term GO:0043473. This protein-coding gene is annotated as a tetraspanin (*tspan36*, see Additional file 1: Table S1). Tetraspanins are a large family of transmembrane proteins (38 homologous genes in vertebrates) that are involved in diverse biological processes, acting as organizers in the membranes of many kinds of animal cells [81]. The functions of all the tetraspanins are not well known, but some members of this gene family have been associated with pigment cell interactions and pigment pattern formation [82]. The *tspan36* gene seems to play an important role in melanocyte biology [83]. Despite spending all or most of their lives in soil, many caecilian species are pigmented, and some are brightly coloured and visually striking, perhaps aposematically in some cases [84], although many are also more drably coloured. Adaptive innovation in *tspan36* might be related to evolutionary changes in pigmentation. Species of *Microcaecilia* have a range of colours and patterns [85] and the ancestral phenotype is currently unclear.

### Other specific traits

Unlike other extant amphibians, many caecilian amphibians have collagenous scales hidden in annular folds in the skin, the function of which is unknown [86, 87]. Some ancestors of caecilians are expected to have had scales over the entire external surface of the body rather than embedded in the skin. The peculiar disposition (when present) of scales within the skin in modern caecilians and their varied patterns of reduction and loss are derived traits plausibly associated with the evolution of a burrowing habit. In extant caecilians, scales are diverse in their number, form and distribution along the body with each of our sampled species presenting a distinct pattern. Collagen chains are structural proteins classified under different types, and they are the main components of skin, connective tissues, bone, teeth and epithelia [88]. We hypothesise that some of the collagen protein-coding genes might code for collagen chains involved in the formation of caecilian scales, particularly *col17a1* that presented a positive selection signature on the Gymnophiona branch and skin tissue specificity expression. We are aware that collagen chains are involved in many other important biological processes, for instance *col4a2* is part of the ECM-receptor interaction pathway on the Gymnophiona branch. Also, one of the candidate collagen genes under positive selection is found in the branch subtending *T. compressicauda* (*col4a1*), a species that lacks annular scales. Mutations in different genes of type IV collagen cause the Alport syndrome in humans, characterized by hearing and eyesight loss among other symptoms [89].

Lipid metabolism and fatty acid metabolism are biological processes associated with several of the genes that bear evidence of positive selection. Lipids have very diverse biological functions and play important roles such as energy storage, signaling, and formation of barriers in the cell membrane. They are also involved in other vital and apomorphic roles in caecilians, including the provision of nutrition to large yolky eggs in oviparous taxa, and to developing fetuses and/or newborns during oviductal and/or skin feeding among teresomatan caecilians [90, 91]. Some of these genes might be related to the synthesis, transformation and/or storage of lipids for these traits. For instance, some of these genes have been found expressed in the yolk of zebrafish (*elovl5*; [92]), in mouse embryoid bodies (*cers6*; [93]), and during vitellogenesis in teleost fish (*cyp17a1*; [94]).

Some of the candidate positively selected genes have different important roles linked to the immune system, for example *tet2* is expressed in T cells [95], *masp1* presents multiple roles in the innate immune response (*masp1*; [96]), *enpp3* regulates allergic responses [97], and *fyn* controls immune receptor signaling status [98]. Innovations in immune system genes within Gymnophiona are unsurprising. The innate amphibian immune system is likely under strong selective pressure, evolving in arms races via interactions with pathogens. The vast majority of adult caecilians live with their bodies in close proximity to moist (probably microbially rich) tropical soil substrates, and it can be expected that the ecomorphological disparity of caecilians relative to their closest relatives and their ecological diversity promoted immunological molecular genetic changes within the group. Amphibians, survivors of the Earth’s last four mass extinction events, are facing an unprecedentedly high risk of extinction that seems to be linked, in part, to challenges to their immune systems [99, 100]. Caecilian conservation biology is very poorly understood [101] and immune system mechanisms are in need of better understanding.

## Conclusions

Molecular adaptive changes in caecilian amphibians are found to be associated mostly with protein-coding gene products with membrane or extracellular location. These genes present low levels of conservation and connectivity (no PPIs and only one functional network were found). The 168 genes that we infer to have been under positive selection are candidate genes with potential to further clarify adaptations of caecilians linked to their unique and variable natural histories. Several of these candidate genes are possibly causally related to differing degrees of fossoriality and hypothesized ecological shifts that might each have led to new ecological opportunities. Experiments (e.g. transfecting cell-lines with a candidate gene and in silico reconstructions of the protein structure) are required to test the function of these protein-coding genes and to identify their particular roles in important processes, such as perception, reduction-oxidation, and aging in caecilians. Functional experiments can be prompted and focused based on genome-wide studies that have narrowed down candidate genes for more thorough investigation. In this study, we identify a set of candidate genes plausibly involved in ecological and evolutionary key processes. Much biological research relies upon a small number of animal models to investigate biological processes but insights from a broader spectrum of organismal diversity, especially from neglected taxa such as caecilians, are also helpful [102].

The inclusion of representatives of additional caecilian lineages in future studies (especially to expand the phylogenetic, ecological, and geographic sampling), and more complete sets of genes from the sampled species (available transcriptome data thus far corresponds mostly to adult animals, [40]) could provide further insights into the selective pressures shaping caecilian molecular evolution. Adaptations are not necessarily only associated with positive selection in protein-coding genes. Changes in regulation can also allow adaptation to new environments, and are thus far unexplored for caecilians. The findings reported here will hopefully provide a foundation for further analyses of the molecular bases of the radiation of Gymnophiona and of molecular evolution in vertebrates more generally.

## Methods

### Genomic data

The source data of this study were the protein-coding gene sequences (both nucleotide and amino-acid level) from reference transcriptomes of five caecilian species (*R. bivittatum*, *C. tentaculata*, *T. compressicauda*, *M. unicolor* and *M. dermatophaga*; assemblies are available from NCBI through BioProject ID number PRJNA387587; [40]) as well as those for the frog *X. tropicalis*, the only amphibian currently represented in the Ensembl database [103]. Species-specific caecilian transcriptomes were de novo assembled from paired-end RNA-seq samples of multiple tissues (kidney, liver, and skin samples for each of the five species plus a selection of other tissues for subsets of the five species: foregut, heart, lung, muscle, spleen, and testis) yielding five reference transcriptomes with a high percentage of completeness. Protein-coding sequences were identified from these assembled sequences with an open reading frame [40]. For each *X. tropicalis* gene, the isoform encoding the longest protein was chosen for analysis, and BLAST searches (blastp tool, version 2.2.28; E-value < 10^− 10^; [104]) were conducted against the proteins of each of the caecilian transcriptomes. Likewise, each caecilian protein sequence was used as a query in a BLAST search against the *X. tropicalis* proteome. Pairs of best reciprocal hits were considered orthologs. Only *X. tropicalis* genes with putative orthologs in all five caecilian species were used in downstream analyses.

For each group of orthologs, the inferred amino acid sequences were aligned using PRANK with default parameters [105]. Given the sensitivity of positive selection analyses to alignment errors, we carried out a thorough filtering of the alignments. First, Gblocks version 0.91b [106] with default settings was used to remove problematic regions. Second, two ad hoc sliding window filters (of 15 and 5 residues) were used to eliminate regions coding for amino acids that are unique to one species (with 10 or more amino acid singletons, or where all five were singletons, respectively; as in as in [107, 108]) because such regions are often associated with annotation or sequencing errors. The resulting amino acid sequence alignments were used to guide the alignment of the corresponding codon sequences.

### Tests of positive selection

To infer positive selection, we performed branch-site model tests [109, 110] for every group of orthologous genes and for every branch of the studied subset of the caecilian phylogeny (based on [40, 69]; Fig. 1), excluding the *X. tropicalis* branch, and computing branch lengths each time for each group of genes using the CODEML program in PAML 4.6 [111]. The branch-site model test (model A vs. null model A) assumes that only a fraction of sites might have undergone positive selection and only along a single a priori identified branch (foreground lineage) on the phylogeny. The test assumes four classes of sites: codons that are conserved (ω < 1), codons that are evolving neutrally (ω = 1), and codons under positive selection (ω > 1) on the foreground branch but conserved (2a) or neutral (2b) on the other (background) branches. Model A was implemented with the default starting value (0.4) for ω (model = 2, NSsites = 2, cleandata = 1 and fix_blength = 0) and used as the alternative hypothesis for the Likelihood Ratio Test (LRT). The null model of the LRTs was the null model A with only one change in the parameters from model A: ω fixed at 1 for sites under positive selection on the foreground branch (2a and 2b sites). *P*-values for the LRTs were computed using the χ^2^ distribution with one degree of freedom, and divided by two [111, 112]. Multiple-testing corrections were conducted following Benjamini and Hochberg’s method in order to control for a false discovery rate (FDR) using R [113]. Genes with a q-value < 0.1 and ω > 1 for the foreground branch (2a and 2b sites) were interpreted as being genes under positive selection. Sites under positive selection were identified by computing posterior probabilities using the Bayes empirical Bayes (BEB) approach [114]. To obtain an estimation of the proportion of tandem complex mutations in our results, we analysed the position of the codons under positive selection from BEB test outputs. Also, the suitability of the positive selection analyses for the multiple sequence alignments of the genes with signatures of selection was tested using the GUIDANCE2 methodology [44], computing the GUIDANCE alignment score for each inference (alignments are available from the Github repository: TorresSanchezM/alignments). The number of genes with signatures of positive selection in sister branches were compared by two-tailed binomial tests under the hypothesis of equal probability of being or not under positive selection (*p* = 0.5) with a 95% confidence level.

### Functional annotation

For each of the putative orthologous groups inferred to be under positive selection, we obtained the associated GO terms from the *X. tropicalis* annotation using the BioMart data-mining tool (Ensembl Genes 95, *Xenopus* genes JGI 4.2; [103]). Novel or uncharacterised genes from *X. tropicalis* were annotated by BLAST searches (blastx tool; [104]) against the Non-redundant protein sequences database. We summarized and visualized the common GO terms of the selected genes and their frequencies of occurrence using REVIGO [43] applying 0.7% allowed similarity (by the semantic similarity method) and using the whole UniProt database [115] to define the size of each GO term. The exploratory REVIGO networks were manually processed to build a unique explanatory plot with general GO categories presented in the REVIGO networks for each of the nine analysed branches. Enrichment analysis for each branch was performed using the GO enrichment analysis tool [116]. Additionally, protein-protein interactions (PPIs) were inferred using STRING [117] with *X. tropicalis* as the reference organism and default settings, comparing the interactions of caecilian protein-coding genes on each branch among themselves to a random set of proteins of similar size, drawn from the chosen genome. Finally, after counting Transcripts Per Million (TPM) units with the RSEM program [118] for each gene under positive selection in the caecilian transcriptomes, gene-expression presence across tissues was determined for cases with more than 5% of the total TPM being found in one tissue type. Tissue specificity was identified when more than 95% of the total TPM were found in one particular tissue type (following [40]).

## Additional file

Additional file 1: **Table S1.** Description of genes inferred to have been under positive selection in caecilian evolution (ω* for sites under positive selection on the foreground branch, 2a and 2b sites; NR database = Non redundant protein database; F = foregut; H = heart; K = kidney; L = liver; Lu = lung; M = muscle; S = skin; Sp = spleen; T = testis). (PDF 300 kb)

## Abbreviations

BEB: Bayes empirical Bayes
dN: Non-synonymous nucleotide substitutions
dS: Synonymous nucleotide substitutions
ECM: Extracellular matrix
FDR: False discovery rate
GO: Gene ontology
LRT: Likelihood ratio test
PPIs: Protein-protein interactions
TPM: Transcripts Per Million
