# Supplementary material for: What lies beneath? Molecular evolution during the radiation of caecilian amphibians
Source: BMC Genomics. 2019 May 9;20:354. doi: 10.1186/s12864-019-5694-1 (PMC6507065; doi:10.1186/s12864-019-5694-1)
Supplement: Supplementary file 1 — Table S1. Description of genes inferred to have been under positive selection in caecilian evolution (ω* for sites under positive selection on the foreground branch, 2a and 2b sites; NR database = Non redundant protein database; F = foregut; H = heart; K = kidney; L = liver; Lu = lung; M = muscle; S = skin; Sp = spleen; T = testis). (PDF 300 kb) [file 12864_2019_5694_MOESM1_ESM.pdf]

Additional file 1: Table S1

What lies beneath? Molecular evolution during the radiation of caecilian amphibians

María Torres-Sánchez[1,2,*] , David J. Gower[3], David Alvarez-Ponce[4], Christopher J. Creevey[5], Mark Wilkinson[3], Diego San Mauro[1]

*Author for Correspondence: torressanchez.maria@gmail.com

Table S1. Description of genes inferred to have been under positive selection in caecilian evolution (ω* for sites under positive selection on the foreground branch, 2a and 2b sites; NR database = Non redundant protein database; F = foregut; H = heart; K = kidney; L = liver; Lu = lung; M = muscle; S = skin; Sp = spleen; T = testis).

**Foreground branch**	**Gene name**	**Gene description**	**GO terms**	**Tissue expression**	***Xenopus* gene ID**	**GUIDANCE2 alignment score**	**ω***	**FDR q-value**
**Gymnophiona**	***acot2***	acyl-CoA thioesterase 2	GO:0005737; GO:0006631; GO:0006637; GO:0016790; GO:0047617	F, H, K, L, Lu, S, T	ENSXETG00000000057	0.961946	107.57442	0.081956824
**Gymnophiona**	***wdr1***	WD repeat domain 1	-	Sp, F, H, K, L, Lu, M, S, T	ENSXETG00000000629	0.996539	999	0.060673056

**Gymnophiona**	***slc34a2***	solute carrier family 34 member 2	GO:0005436; GO:0005737; GO:0005886; GO:0005903; GO:0015321; GO:0016020; GO:0016021; GO:0016324; GO:0030643; GO:0031982; GO:0035435; GO:0035725; GO:0044341	K, Lu, S	ENSXETG00000000954	0.996627	8.33671	0.081956824
**Gymnophiona**	***sod3***	superoxide dismutase 3	GO:0004784; GO:0005507; GO:0005615; GO:0005737; GO:0006801; GO:0008270; GO:0016491; GO:0019430; GO:0046872; GO:0055114	H, K, Lu, S, T	ENSXETG00000002122	0.991515	143.96716	0.075170199
**Gymnophiona**	***col4a2***	collagen type IV alpha 2 chain	GO:0005201; GO:0005576; GO:0005578; GO:0005581; GO:0005604	Sp, F, H, K, Lu, M, T	ENSXETG00000002635	0.984184	98.85225	0.082470139
**Gymnophiona**	***akr1a1***	aldo-keto reductase family 1 member A1	GO:0008106; GO:0016491; GO:0055114	Sp, F, H, K, L, Lu, M, S, T	ENSXETG00000003499	0.999892	300.07976	0.060673056
**Gymnophiona**	***als2cl***	ALS2 C-terminal like	-	F, K, Lu, S, T	ENSXETG00000003686	0.997792	54.6686	0.050457662
**Gymnophiona**	***nup155***	nucleoporin 155kDa	GO:0000972; GO:0005643; GO:0006405; GO:0006606; GO:0006913; GO:0017056; GO:0036228; GO:0044611	Sp, F, H, K, L, Lu, M, S, T	ENSXETG00000004785	0.998422	40.12923	0.081956824
**Gymnophiona**	***c10orf35***	chromosome 10 open reading frame 35	GO:0016020; GO:0016021	Sp, F, H, K, L, Lu, M, S, T	ENSXETG00000006461	0.997727	418.15868	0.082538498

**Gymnophiona**	***ddx17***	DEAD-box helicase 17	GO:0000166; GO:0003676; GO:0004004; GO:0004386; GO:0005524; GO:0010501; GO:0016787; GO:0045893	Sp, F, H, K, L, Lu, S, T	ENSXETG00000006900	1	999	0.035291841
**Gymnophiona**	***adamts7***	ADAM metallopeptidase with thrombospondin type 1 motif 7	GO:0004222; GO:0005578; GO:0006508; GO:0008233; GO:0008237; GO:0008270; GO:0031012; GO:0046872	K, L, Lu, S, T	ENSXETG00000007838	0.997315	22.57471	0.045769604
**Gymnophiona**	***sult1c1***	uncharacterised in Ensembl database; Sulfotransferase 1C1-like protein (from NR database)	GO:0008146; GO:0016740	F, K, L, Lu, S	ENSXETG00000009265	0.993079	87.87366	0.050457662
**Gymnophiona**	***nckipsd***	NCK interacting protein with SH3 domain	-	Sp, F, H, K, L, Lu, M, S, T	ENSXETG00000009319	0.995934	59.17284	0.088559456
**Gymnophiona**	***esyt1***	extended synaptotagmin-like protein 1	GO:0008289; GO:0016020; GO:0016021; GO:0031227; GO:0044232	Sp, F, H, K, L, Lu, S, T	ENSXETG00000009481	0.996099	55.12752	0.021371019
**Gymnophiona**	***msn***	moesin	GO:0003779; GO:0005737; GO:0005856; GO:0008092; GO:0019898	Sp, F, H, K, L, Lu, S, T	ENSXETG00000009770	0.999842	4.80253	0.075170199
**Gymnophiona**	***aqp9***	aquaporin 9	GO:0005215; GO:0005372; GO:0006833; GO:0015105; GO:0015168; GO:0015204; GO:0015698; GO:0015793; GO:0016020;	L, Lu, M, S	ENSXETG00000010861	0.997696	999	0.03351781

			GO:0016021; GO:0071918					
**Gymnophiona**	***slc22a31***	solute carrier family 22 member 31	GO:0008514; GO:0015711; GO:0016020; GO:0016021; GO:0022857; GO:0055085	F, K, L, Lu, S	ENSXETG00000011276	0.996889	178.82387	0.071653906
**Gymnophiona**	***rph3a***	rabphilin 3A	GO:0005509; GO:0005544; GO:0005886; GO:0006886; GO:0006906; GO:0016020; GO:0017137; GO:0017158; GO:0019905; GO:0030276; GO:0046872; GO:0048791; GO:0070382; GO:0098793	K, Lu, T	ENSXETG00000011467	1	999	3.09E-36
**Gymnophiona**	***lamc1***	laminin subunit gamma 1	GO:0001654; GO:0005604; GO:0007411; GO:0007420; GO:0007517; GO:0007519; GO:0007634; GO:0030903; GO:0031290; GO:0048570; GO:0048731; GO:0048854; GO:0050908; GO:0061053; GO:0070831	Sp, F, H, K, Lu, M, T	ENSXETG00000012525	0.998305	23.15197	0.060673056
**Gymnophiona**	***tet2***	tet methylcytosine dioxygenase 2	GO:0030097; GO:0030099; GO:0030218; GO:0060319; GO:0070989; GO:0098508	Sp, F, K, L, Lu, M, S	ENSXETG00000014101	0.99575	100.54979	0.081956824
**Gymnophiona**	***gstcd***	glutathione S-transferase C-terminal	GO:0005737	Sp, F, K, L, Lu, S, T	ENSXETG00000014108	0.99632	131.16589	0.081956824

		domain						
**Gymnophiona**	***nup153***	nucleoporin 153kDa	GO:0001525; GO:0005622; GO:0006405; GO:0006606; GO:0008139; GO:0008270; GO:0017056; GO:0046872	F, K, Lu, M, S, T	ENSXETG00000014197	0.991718	999	0.045769604
**Gymnophiona**	***gdpd5***	glycerophosphodiester phosphodiesterase domain containing 5	GO:0006629; GO:0008081; GO:0008889; GO:0016020; GO:0016021	Sp, H, L, Lu, M, S, T	ENSXETG00000015053	0.996516	31.09229	0.082470139
**Gymnophiona**	***tacc2***	transforming acidic coiled-coil containing protein 2	GO:0000226; GO:0005737; GO:0008283; GO:0015630; GO:0021987	F, H, Lu, M, S, T	ENSXETG00000015587	0.989159	103.81618	0.081956824
**Gymnophiona**	***klhdc10***	kelch domain containing 10	-	Sp, F, H, K, L, Lu, M, S, T	ENSXETG00000016301	1	56.55156	0.066797583
**Gymnophiona**	***golga1***	golgin A1	GO:0005794	F, H, K, Lu, M, S, T	ENSXETG00000016840	0.997726	50.29481	0.042081125
**Gymnophiona**	***pigr***	polymeric immunoglobulin receptor	GO:0016020; GO:0016021	F, K, L, Lu, S	ENSXETG00000017102	0.988724	54.9727	0.081956824
**Gymnophiona**	***gigyf1***	GRB10 interacting GYF protein 1	-	F, K, L, Lu, S	ENSXETG00000018415	0.98933	63.29151	0.000103512
**Gymnophiona**	***cul9***	cullin 9	GO:0006511; GO:0008270; GO:0031625; GO:0046872	Sp, F, K, L, Lu, S, T	ENSXETG00000018504	0.998546	47.26087	0.071653906
**Gymnophiona**	***cdhr2***	cadherin related family member 2	GO:0005509; GO:0005886; GO:0007155; GO:0007156; GO:0016020; GO:0016021	Sp, F, K, Lu, T	ENSXETG00000019629	0.991436	52.04677	0.00251902
**Gymnophiona**	***hprt1***	hypoxanthine phosphoribosyltransferase 1	GO:0009116	F, K, L, Lu, M, S, T	ENSXETG00000019768	1	19.00688	0.081956824
**Gymnophiona**	***cgn***	cingulin	GO:0003774; GO:0016459	F, K, L, Lu, S, T	ENSXETG00000020726	0.996984	39.28213	0.045769604
**Gymnophiona**	***itga3***	integrin	GO:0007155;	F, H, K, L,	ENSXETG00000021920	0.997466	32.10395	0.045769604

		subunit alpha 3	GO:0007229; GO:0008305; GO:0016020; GO:0016021	Lu, S, T				
**Gymnophiona**	***p2ry11***	purinergic receptor P2Y G-protein coupled 11	GO:0001973; GO:0004871; GO:0004930; GO:0007165; GO:0007186; GO:0007200; GO:0016020; GO:0016021; GO:0023041; GO:0035589; GO:0045028; GO:0045031	Sp, K, L, Lu, T	ENSXETG00000022059	0.985115	40.28705	0.045769604
**Gymnophiona**	***ptprh***	protein tyrosine phosphatase receptor type H	GO:0004725; GO:0006470; GO:0016020; GO:0016021; GO:0016311; GO:0016791; GO:0035335	F, K, L, Lu, S	ENSXETG00000022920	0.997601	184.64403	0.021371019
**Gymnophiona**	***spen***	spen family transcriptional repressor	GO:0000166; GO:0000398; GO:0003676; GO:0005634	Sp, F, H, K, L, Lu, M, S, T	ENSXETG00000023114	0.996525	64.05463	0.081956824
**Gymnophiona**	***qsox1***	quiescin sulfhydryl oxidase 1	GO:0003756; GO:0005615; GO:0016020; GO:0016021; GO:0016491; GO:0016971; GO:0016972; GO:0030173; GO:0045454; GO:0055114	F, L, S, T	ENSXETG00000023156	0.990129	105.32195	0.021371019
**Gymnophiona**	***vwf***	von Willebrand factor	GO:0005578; GO:0007155; GO:0007596; GO:0007599	F, H, K, Lu	ENSXETG00000023591	0.996418	999	0.060673056
**Gymnophiona**	***cdk12***	cyclin-dependent kinase 12	GO:0004672; GO:0005524; GO:0006468	Sp, F, K, L, Lu, S, T	ENSXETG00000023695	1	4.32061	0.060673056
**Gymnophiona**	***tbrg4***	transforming growth factor	GO:0004672; GO:0006468	Sp, F, H, K, L, Lu, M,	ENSXETG00000023866	0.999515	999	0.057479166

		beta regulator 4		S, T				
**Gymnophiona**	***tcf19***	transcription factor 19	-	Sp, F, K, Lu, M, S, T	ENSXETG00000024079	0.979246	999	0.081956824
**Gymnophiona**	***spg11***	spatacsin vesicle trafficking associated	GO:0007399; GO:0007409	Sp, F, H, K, L, Lu, M, S, T	ENSXETG00000025297	0.994661	93.96152	0.077360076
**Gymnophiona**	***rps13***	ribosomal protein S13	GO:0003735; GO:0005730; GO:0005840; GO:0006412; GO:0022627; GO:0070181	Sp, F, H, K, L, Lu, M, S, T	ENSXETG00000026454	0.980159	999	0.081956824
**Gymnophiona**	***gsto2***	glutathione S-transferase omega 2	GO:0004364; GO:0005737; GO:0008152	F, K, L, M, T	ENSXETG00000026602	0.992991	204.42538	0.082470139
**Gymnophiona**	***tnrc6a***	trinucleotide repeat containing 6A	GO:0000166; GO:0000289; GO:0003676; GO:0017148; GO:0035278	Sp, F, K, L, Lu, S, T	ENSXETG00000030437	0.999903	7.70315	0.069829147
**Gymnophiona**	***cp***	ceruloplasmin	GO:0004322; GO:0005507; GO:0005634; GO:0005737; GO:0006825; GO:0006879; GO:0016491; GO:0046872; GO:0055114	H, L	ENSXETG00000031159	0.997173	61.27401	0.082470139
**Gymnophiona**	***acadvl***	acyl-CoA dehydrogenase very long chain	GO:0003995; GO:0008152; GO:0016491; GO:0016627; GO:0050660; GO:0055114; GO:0060322	Sp, F, H, K, L, Lu, M, S, T	ENSXETG00000031271	1	87.2058	0.001074924
**Gymnophiona**	***col16a1***	uncharacterised in Ensembl database; collagen type XVI alpha 1 chain (from NR database)	GO:0005615; GO:0005178; GO:0007155; GO:0031012; GO:0005201; GO:0030198; GO:0033627; GO:0051894	Sp, F, H, K, Lu, M, S, T	ENSXETG00000033245	0.994107	999	0.077360076

**Gymnophiona**	***col17a1***	collagen type XVII alpha 1 chain	GO:0005578; GO:0016020; GO:0016021	S	ENSXETG00000033563	0.997395	136.30841	0.009786183
**Gymnophiona**	***slc18a1***	uncharacteris-ed in Ensembl database; chromaffin granule amine transporter (from NR database)	GO:0005887; GO:0006837; GO:0015222; GO:0015842; GO:0016020; GO:0016021; GO:0055085; GO:0098793	F, K, S, T	ENSXETG00000033569	0.996987	139.35786	0.045769604
**Teresomata**	***fam3b***	family with sequence similarity 3 member B	-	F, K, L, Lu, S	ENSXETG00000005180	0.992889	998.98757	0.058383372
**Teresomata**	***aoc3***	amine oxidase copper containing 3	GO:0005507; GO:0007601; GO:0008131; GO:0009308; GO:0016020; GO:0016021; GO:0016491; GO:0046872; GO:0048038; GO:0055114	F, K, L, Lu, M	ENSXETG00000012588	0.997341	998.99982	0.068228787
**Teresomata**	***mbd5***	methyl-CpG binding domain protein 5	GO:0003677; GO:0003682; GO:0005634; GO:0010369	Sp, F, H, K, L, Lu, M, S, T	ENSXETG00000018214	0.997288	998.99978	0.049582247
**Teresomata**	***hgs***	hepatocyte growth factor-regulated tyrosine kinase substrate	GO:0005622; GO:0006886; GO:0046872	Sp, F, H, K, L, Lu, M, S, T	ENSXETG00000019701	0.99836	999	0.039962285
**Teresomata**	***masp1***	mannan-binding lectin serine peptidase 1	GO:0001755; GO:0001867; GO:0004252; GO:0005509; GO:0005576; GO:0005615; GO:0005737; GO:0006508; GO:0006956; GO:0008233; GO:0008236;	K, L, T	ENSXETG00000019757	0.975646	482.51659	2.34E-22

			GO:0016787; GO:0046872					
**Teresomata**	***pcdh7***	protocadherin 7	GO:0005509; GO:0005886; GO:0007155; GO:0007156; GO:0016020; GO:0016021	Sp, F, H, K, Lu, S, T	ENSXETG00000022281	0.999992	999	0.032846913
**Teresomata**	***tnc***	tenascin C	GO:0007155; GO:0031012; GO:0042127	F, K, Lu, M, S	ENSXETG00000023938	0.999483	557.19204	3.94E-05
**Teresomata**	***sypl1***	synaptophysin-like protein 1	GO:0005215; GO:0008021; GO:0016020; GO:0016021; GO:0030285	Sp, F, K, L, Lu, M, S, T	ENSXETG00000025677	0.992631	999	0.039962285
Rhinatrema bivittatum	***plpp1***	phospholipid phosphatase 1	GO:0005886; GO:0005887; GO:0006629; GO:0006644; GO:0007165; GO:0008195; GO:0016020; GO:0016021; GO:0016311; GO:0042577; GO:0046839	Sp, F, H, K, L, Lu, M, S, T	ENSXETG00000000375	0.999783	998.96886	0.087167945
Rhinatrema bivittatum	***mtg2***	mitochondrial ribosome-associated GTPase 2	GO:0000287; GO:0003924; GO:0005525	Sp, F, H, K, L, Lu, M, S, T	ENSXETG00000002001	0.998786	38.67929	0.034033894
Rhinatrema bivittatum	***clic3***	chloride intracellular channel 3	GO:0005254; GO:0006821; GO:1902476	F, Lu, S, T	ENSXETG00000003974	0.99807	998.98992	0.05538756
Rhinatrema bivittatum	***cenpa***	centromere protein A	GO:0000775; GO:0000776; GO:0000777; GO:0000786; GO:0003677; GO:0005634; GO:0005694; GO:0046982	Sp, S, T	ENSXETG00000005197	0.999214	999	0.05538756
Rhinatrema bivittatum	***atp1a2***	ATPase Na+/K+ transporting alpha 2	GO:0000166; GO:0001947; GO:0001966; GO:0005391;	F, H, K, Lu, M, S, T	ENSXETG00000008125	0.999961	48.43624	0.079634778

		polypeptide	GO:0005524; GO:0005623; GO:0006811; GO:0006813; GO:0006814; GO:0007368; GO:0007507; GO:0007519; GO:0010248; GO:0016020; GO:0016021; GO:0016787; GO:0046872; GO:0051480; GO:0060047; GO:0061371;					
Rhinatrema bivittatum	***rcn1***	reticulocalbin 1	GO:0005509	Sp, F, K, L, Lu, M, S, T	ENSXETG00000008174	0.995089	58.64572	0.087167945
Rhinatrema bivittatum	***nuf2***	NUF2; NDC80 kinetochore complex component	GO:0000775; GO:0007507	Sp, H, K, Lu, M, T	ENSXETG00000010463	0.993007	11.1057	0.087167945
Rhinatrema bivittatum	***col5a2***	collagen type V alpha 2 chain	GO:0005201	Sp, F, H, K, L, Lu, M, S, T	ENSXETG00000010784	0.998895	259.99922	0.011840021
Rhinatrema bivittatum	***rpl13a***	ribosomal protein L13a	GO:0003729; GO:0003735; GO:0005840; GO:0006412; GO:0015934; GO:0022625; GO:0030529	Sp, F, K, L, Lu, M, S, T	ENSXETG00000014144	1	5.33779	7.96E-15
Rhinatrema bivittatum	***rcc2***	regulator of chromosome condensation 2	GO:0001755	Sp, F, K, Lu, S, T	ENSXETG00000014793	0.999976	998.99973	0.032134195
Rhinatrema bivittatum	***anxa2***	annexin A2	GO:0004859; GO:0005509; GO:0005544; GO:0008092; GO:0043086	F, H, K, L, Lu, M, S, T	ENSXETG00000015289	0.998053	83.87076	0.08170457
Rhinatrema bivittatum	***anxa6***	annexin A6	GO:0001778; GO:0005509; GO:0005544	Sp, F, H, Lu, M, T	ENSXETG00000015832	0.998564	23.63599	0.067225054
Rhinatrema	***hdgf***	hepatoma-	-	Sp, F, K, L,	ENSXETG00000018516	0.998676	99.10972	0.072192303

bivittatum		derived growth factor		Lu, M, S, T				
Rhinatrema bivittatum	***yipf1***	Yip1 domain family member 1	GO:0005794; GO:0016020; GO:0016021; GO:0017137	Sp, F, H, K, L, Lu, M, S, T	ENSXETG00000019983	0.998483	999	0.000519394
Rhinatrema bivittatum	***tbc1d31***	TBC1 domain family member 31	-	Sp, F, H, K, L, Lu, M, S, T	ENSXETG00000023189	0.997757	310.36313	0.037596663
Rhinatrema bivittatum	***parp14.2***	poly (ADP-ribose) polymerase family member 14 gene 2	GO:0000166; GO:0003676; GO:0003950; GO:0016740; GO:0016757	Sp, F, H, K, L, Lu, M, S, T	ENSXETG00000023399	0.986106	827.88309	0.08170457
Rhinatrema bivittatum	***tnc***	tenascin C	GO:0007155; GO:0031012; GO:0042127	F, K, Lu, M, S	ENSXETG00000023938	0.999483	105.06599	0.064561005
Caecilia + Typhlonectes	***aqp3***	aquaporin 3	GO:0005215; GO:0016020; GO:0016021	F, K, Lu, M, S	ENSXETG00000002151	1	998.99918	0.094399642
Caecilia+ Typhlonectes	***fadd***	Fas associated via death domain	GO:0007165; GO:0042981; GO:0043065	F, H, K, L, Lu, M, S, T	ENSXETG00000003799	0.989481	52.29035	0.050859898
Caecilia + Typhlonectes	***efemp1***	EGF containing fibulin-like extracellular matrix protein 1	GO:0005006; GO:0005509; GO:0007173; GO:0031012	F, H, K, Lu	ENSXETG00000006076	0.997067	14.04564	0.007001
Caecilia + Typhlonectes	***utp14a***	UTP14A small subunit processome component	GO:0005730; GO:0006364; GO:0030490; GO:0032040	Sp, F, H, K, L, Lu, M, S, T	ENSXETG00000007465	0.996442	22.93783	0.094399642
Caecilia + Typhlonectes	***parp9***	poly(ADP-ribose) polymerase family member 9	GO:0003950	F, H, K, L, Lu, S, T	ENSXETG00000007985	0.99344	999	0.094399642
Caecilia + Typhlonectes	***enpp3***	ectonucleotide pyrophosphatase/ phosphodiesterase 3	GO:0003676; GO:0003824; GO:0004528; GO:0004551; GO:0005044; GO:0006898; GO:0006955;	Sp, F, K, L, Lu, M, S, T	ENSXETG00000008244	0.998141	998.99977	0.094399642

			GO:0008152; GO:0016020; GO:0016021; GO:0016787; GO:0030247; GO:0046872; GO:0090305					
Caecilia + Typhlonectes	***ybx1***	Y-box binding protein 1	GO:0003676; GO:0003677; GO:0003723; GO:0006355; GO:0008190; GO:0045947; GO:0048025; GO:0050686; GO:0051236; GO:1900364	Sp, F, H, K, L, Lu, M, S, T	ENSXETG00000013436	1	999	0.050859898
Caecilia + Typhlonectes	***acat1***	acetyl-CoA acetyltransferase 1	GO:0003824; GO:0008152; GO:0016740; GO:0016746; GO:0016747	F, H, K, L, M, S, T	ENSXETG00000014477	1	999	0.094399642
Caecilia + Typhlonectes	***axl***	AXL receptor tyrosine kinase	GO:0000166; GO:0004672; GO:0004713; GO:0005524; GO:0006468; GO:0016020; GO:0016021; GO:0016301; GO:0016310; GO:0016740; GO:0018108	F, K, Lu, S, T	ENSXETG00000018708	0.992414	41.86018	0.053497776
Caecilia + Typhlonectes	***pfn2***	profilin 2	GO:0003779; GO:0030036; GO:0030833	F, H, K, L, Lu, M, T	ENSXETG00000020090	0.994974	999	6.92E-05
Caecilia + Typhlonectes	***exog***	endo/ exonuclease (5'-3'); endonuclease G-like	GO:0003676; GO:0016787; GO:0046872	Sp, F, K, L, Lu, M, S, T	ENSXETG00000021000	0.999065	999	0.094399642
Caecilia + Typhlonectes	***tmem27***	transmembrane protein 27	GO:0006508; GO:0008237; GO:0008241; GO:0016020; GO:0016021	K	ENSXETG00000022466	0.996767	999	0.01469052

Caecilia + Typhlonectes	***scarb2***	scavenger receptor class B member 2	GO:0004872; GO:0005764; GO:0016020; GO:0016021	Sp, F, H, K, L, Lu, M, S, T	ENSXETG00000024116	0.996537	53.04016	0.004245511
Caecilia + Typhlonectes	***itgb1***	integrin subunit beta 1	GO:0004872; GO:0007155; GO:0007160; GO:0007229; GO:0008305; GO:0016020; GO:0016021	Sp, F, H, K, Lu, M, S, T	ENSXETG00000026716	0.99625	376.16418	0.006565164
Caecilia + Typhlonectes	***ano9***	uncharacteris-ed in Ensembl database; anoctamin-9-like protein (from NR database)	GO:0016020; GO:0016021; GO:0046983; GO:0061588	F, K, Lu, S	ENSXETG00000031447	0.996781	217.23189	0.094399642
Caecilia tentaculata	***dsc3***	desmocollin 3	GO:0002159; GO:0005509; GO:0005886; GO:0007155; GO:0007156; GO:0007507; GO:0016020; GO:0016021; GO:0055113; GO:0060027	F, S, T	ENSXETG00000004721	0.991111	21.38483	0.019034822
Caecilia tentaculata	***agl***	*amylo-alpha-1, 6-glucosidase, 4-alpha-glucanotransferase*	GO:0004134; GO:0004135; GO:0003824; GO:0005978; GO:0005980	H, K, L, M, S	ENSXETG00000013185	0.999656	11.74808	0.011498941
Caecilia tentaculata	***ppil4***	peptidylprolyl isomerase like 4	GO:0000166; GO:0000413; GO:0003676; GO:0003755; GO:0006457	Sp, F, H, K, L, Lu, S, T	ENSXETG00000021385	0.998125	999	0.052032424
Caecilia tentaculata	***pfkp***	phosphofructo kinase; platelet	GO:0003872; GO:0005524; GO:0005737; GO:0006002; GO:0006096; GO:0061615	Sp, F, H, K, L, Lu, S, T	ENSXETG00000021922	0.997169	998.99901	2.13E-08

Caecilia tentaculata	***tubgcp6***	tubulin gamma complex associated protein 6	GO:0000226; GO:0000922; GO:0000923; GO:0005200; GO:0005737; GO:0005813; GO:0005815; GO:0005856; GO:0005874; GO:0007020; GO:0008274; GO:0031122; GO:0043015; GO:0051011; GO:0051298; GO:0051415; GO:0090307	Sp, F, H, K, L, Lu, M, S, T	ENSXETG00000022264	0.99996	998.99915	0.019034822
Caecilia tentaculata	***man2a1***	mannosidase; alpha class 2A member 1	GO:0000139; GO:0003824; GO:0004553; GO:0004559; GO:0005975; GO:0006013; GO:0006491; GO:0006517; GO:0008270; GO:0016020; GO:0016021; GO:0030246	Sp, F, K, L, Lu, S, T	ENSXETG00000026530	0.997602	998.9581	7.40E-13
Caecilia tentaculata	***trmt10c***	tRNA methyltransferase 10C	GO:0005739; GO:0008033	Sp, F, H, K, L, Lu, M, S, T	ENSXETG00000029921	0.984885	16.75471	0.071059442
Typhlonectes compressicauda	***f2***	coagulation factor 2 thrombin	GO:0004252; GO:0005509; GO:0005576; GO:0006508; GO:0007596; GO:0008233; GO:0008236; GO:0016787	L	ENSXETG00000001982	0.992934	39.56098	0.082333407
Typhlonectes compressicauda	***col4a1***	collagen type IV alpha 1	GO:0005201; GO:0005576; GO:0005578; GO:0005581; GO:0005604	Sp, F, H, K, Lu, M, T	ENSXETG00000002637	0.987688	30.19389	0.082333407
Typhlonectes	***slc30a10***	solute carrier	GO:0005385;	F, K, L, Lu,	ENSXETG00000002721	0.990615	50.94049	0.026827163

compressicauda		family 30 member 10	GO:0005886; GO:0006812; GO:0008324; GO:0010043; GO:0016020; GO:0016021; GO:0055085; GO:0061088; GO:0071577; GO:0098655	S, T				
Typhlonectes compressicauda	***camkmt***	calmodulin-lysine N-methyltransferase	GO:0005737; GO:0018022; GO:0018025	Sp, F, H, K, L, Lu, M, S, T	ENSXETG00000002754	0.997654	268.68298	0.095269185
Typhlonectes compressicauda	***klkb1***	kallikrein B1	GO:0004252; GO:0005576; GO:0006508	L	ENSXETG00000005867	0.991541	12.67365	0.095269185
Typhlonectes compressicauda	***mios***	missing oocyte meiosis regulator homolog	-	Sp, F, H, K, L, Lu, M, S, T	ENSXETG00000007293	0.999266	889.60098	0.072501743
Typhlonectes compressicauda	***polr2a***	polymerase RNA II	GO:0001055; GO:0003677; GO:0003899; GO:0005665; GO:0006351; GO:0006366; GO:0016740; GO:0016779	F, H, K, L, Lu, S, T	ENSXETG00000012465	1	998.99995	0.000307004
Typhlonectes compressicauda	***prkag3***	protein kinase AMP-activated gamma 3 non-catalytic subunit	GO:0016301; GO:0016310	H, M, S	ENSXETG00000013879	0.995087	998.99942	0.062065169
Typhlonectes compressicauda	***cwc22***	CWC22 homolog spliceosome-associated protein	GO:0000398; GO:0003723; GO:0071006; GO:0071013	Sp, F, H, K, L, Lu, M, S, T	ENSXETG00000014099	0.985994	114.97026	0.030115536
Typhlonectes compressicauda	***ate1***	arginyltransferase 1	GO:0004057; GO:0005737; GO:0016598; GO:0016740; GO:0016746	Sp, F, H, K, L, Lu, M, S, T	ENSXETG00000015591	0.991817	998.99868	0.035809617
Typhlonectes compressicauda	***myh4***	myosin heavy chain 3 embryonic	GO:0000166; GO:0003774; GO:0003779;	H, M, S	ENSXETG00000016248	0.997726	998.99998	0.003513682

		skeletal muscle	GO:0005524; GO:0016459					
Typhlonectes compressicauda	***thoc5***	THO complex 5	-	Sp, F, H, K, L, Lu, M, S, T	ENSXETG00000016419	1	998.99992	0.095269185
Typhlonectes compressicauda	***arhgap33***	Rho GTPase activating protein 33	GO:0005096; GO:0005938; GO:0007165; GO:0007264; GO:0015629; GO:0035091; GO:0043547	Sp, F, H, K, L, Lu, S, T	ENSXETG00000017543	0.998082	999	0.095269185
Typhlonectes compressicauda	***fr47***	uncharacteris-ed in Ensembl database; freeze-responsive liver protein (from NR database)	GO:0001775; GO:0001971; GO:0005576	H, L	ENSXETG00000018913	0.924565	999	0.05271803
Typhlonectes compressicauda	***clcn3***	chloride channel voltage-sensitive 3	GO:0005216; GO:0005247; GO:0005623; GO:0005887; GO:0006811; GO:0006821; GO:0016020; GO:0016021; GO:0034220; GO:0045794; GO:0055085; GO:0072320; GO:1902476	Sp, F, K, L, Lu, S, T	ENSXETG00000023146	1	998.99997	0.030115536
Typhlonectes compressicauda	***fam13a***	family with sequence similarity 13 member A	GO:0007165	Sp, F, K, Lu, M, S, T	ENSXETG00000023661	0.999469	273.35518	0.086409921
Typhlonectes compressicauda	***adgrg6***	adhesion G protein-coupled receptor G6	GO:0004888; GO:0004930; GO:0007166; GO:0007186; GO:0016020; GO:0016021	Sp, K, L, Lu, M, S, T	ENSXETG00000030163	0.997114	269.96578	0.086409921
Typhlonectes compressicauda	***dsg2***	desmoglein 2	GO:0005509; GO:0005886; GO:0007155;	F, K, L, Lu, S, T	ENSXETG00000034243	0.994103	16.30646	0.035809617

			GO:0007156; GO:0016020; GO:0016021					
Microcaecilia	***pinx1***	PIN2/TERF1 interacting telomerase inhibitor 1	GO:0003676; GO:0005730; GO:0010521; GO:0051974	Sp, F, H, K, L, Lu, M, S, T	ENSXETG00000000688	0.989023	109.0809	0.00136397
Microcaecilia	***col4a2***	collagen; type IV alpha 2	GO:0005201; GO:0005576; GO:0005578; GO:0005581; GO:0005604	Sp, F, H, K, Lu, M, T	ENSXETG00000002635	0.984184	56.26914	0.052045712
Microcaecilia	***fam3b***	family with sequence similarity 3 member B	-	F, K, L, Lu, S	ENSXETG00000005180	0.992889	67.2091	0.002717397
Microcaecilia	***iqsec2***	IQ motif and Sec7 domain 2	GO:0005086; GO:0030036; GO:0032012; GO:0043547	Sp, F, H, K, Lu, M, T	ENSXETG00000007177	1	1.86049	5.17E-41
Microcaecilia	***ddx24***	DEAD-box helicase 24	GO:0000166; GO:0003676; GO:0004004; GO:0004386; GO:0005524; GO:0010501; GO:0016787	Sp, F, H, K, L, Lu, M, S, T	ENSXETG00000010314	0.992942	70.67374	0.089979786
Microcaecilia	***mrps7***	mitochondrial ribosomal protein S7	GO:0006412	Sp, F, H, K, L, Lu, M, S, T	ENSXETG00000012510	0.995197	999	0.052045712
Microcaecilia	***elovl5***	ELOVL fatty acid elongase 5	GO:0005783; GO:0005789; GO:0006629; GO:0006631; GO:0006633; GO:0006636; GO:0009922; GO:0016020; GO:0016021; GO:0016740; GO:0019367; GO:0019368; GO:0030425; GO:0030497; GO:0042759; GO:0042761;	F, K, L, Lu, T	ENSXETG00000015994	0.99995	742.62964	0.058323282

			GO:0042995; GO:0043025; GO:0097447; GO:0102336; GO:0102337; GO:0102338					
Microcaecilia	***ca5b***	mitochondrial carbonic anhydrase VB	GO:0004089; GO:0005739; GO:0006730; GO:0008270; GO:0046872; GO:2000021	F, K, L, Lu, S	ENSXETG00000016594	0.995546	95.57838	0.00136397
Microcaecilia	***yes1***	YES proto-oncogene 1 Src family tyrosine kinase	GO:0000166; GO:0004672; GO:0004713; GO:0004715; GO:0005102; GO:0005524; GO:0006468; GO:0007169; GO:0016301; GO:0016310; GO:0016477; GO:0016740; GO:0030154; GO:0031234; GO:0034334; GO:0038083; GO:0042127; GO:0045087; GO:0045859; GO:0046777; GO:0060027	F, H, K, L, Lu, S, T	ENSXETG00000019176	1	2.81761	8.07E-05
Microcaecilia	***basp1***	brain abundant membrane attached signal protein 1	-	Sp, F, K, L, Lu, M, S, T	ENSXETG00000021380	0.999947	999	0.021733743
Microcaecilia	***tspan36***	tetraspanin 36	GO:0005887; GO:0007166; GO:0016020; GO:0016021; GO:0043473	Sp, F, K, L, Lu, T	ENSXETG00000022371	0.999233	543.60023	0.052045712
Microcaecilia	***acp1***	acid phosphatase 1	GO:0003993; GO:0004725;	Sp, F, H, K, L, Lu, M,	ENSXETG00000027987	0.999933	366.58784	0.052045712

			GO:0004726; GO:0005737; GO:0006470; GO:0035335	S, T				
Microcaecilia	***plg***	uncharacteris-ed in Ensembl database; plasminogen (from NR database)	GO:0004252; GO:0006508; GO:0008233; GO:0008236; GO:0016787	L	ENSXETG00000033306	0.998386	998.99948	0.022116997
Microcaecilia unicolor	***col12a1***	collagen type XII alpha 1	GO:0005615	K, L, Lu, M, S, T	ENSXETG00000003603	0.995985	136.70977	9.56E-35
Microcaecilia unicolor	***cat2***	catalase gene 2	GO:0004096; GO:0004601; GO:0005739; GO:0005777; GO:0006979; GO:0016491; GO:0020037; GO:0042542; GO:0042744; GO:0046872; GO:0055114; GO:0098869	F, K, L	ENSXETG00000003981	0.994703	56.00761	0.000183547
Microcaecilia unicolor	***fabp2***	intestinal fatty acid binding protein 2	GO:0005215; GO:0005504; GO:0008289	F, M, S	ENSXETG00000004045	0.9946	999	0.002260627
Microcaecilia unicolor	***lamp2***	lysosomal-associated membrane protein 2	GO:0005764; GO:0005765; GO:0016020; GO:0016021	F, H, K, L, Lu, M, S, T	ENSXETG00000004476	0.984011	998.9942	0.001628235
Microcaecilia unicolor	***dhx36***	DEAH-box helicase 36	GO:0000166; GO:0003676; GO:0004004; GO:0004386; GO:0005524; GO:0005737; GO:0006396; GO:0008026; GO:0016787	Sp, F, H, K, L, Lu, S, T	ENSXETG00000007768	1	27.42023	0.067761385
Microcaecilia unicolor	***sptlc3***	serine palmitoyltrans ferase long chain base subunit 3	GO:0003824; GO:0008152; GO:0009058; GO:0016020; GO:0016021; GO:0016740;	Sp, F, H, K, L, Lu, S, T	ENSXETG00000008083	0.99893	296.87483	0.002260627

			GO:0030170					
Microcaecilia unicolor	***erbb3***	erb-b2 receptor tyrosine kinase 3	GO:0000166; GO:0004672; GO:0004714; GO:0004716; GO:0005524; GO:0005622; GO:0006468; GO:0007169; GO:0016020; GO:0016021; GO:0018108; GO:0023014; GO:0035556	F, K, Lu, S, T	ENSXETG00000009463	0.993945	15.59687	0.087485616
Microcaecilia unicolor	***pih1d2***	PIH1 domain containing 2		F, H, K, L, Lu, S, T	ENSXETG00000010194	0.999678	658.6695	0.01025015
Microcaecilia unicolor	***col5a2***	collagen type V alpha 2 chain	GO:0005201	Sp, F, H, K, L, Lu, M, S, T	ENSXETG00000010784	0.998895	999	0.014683217
Microcaecilia unicolor	***tarbp2***	TAR RNA binding protein 2	GO:0003723; GO:0003725; GO:0005737; GO:0006417; GO:0016442; GO:0030422; GO:0030423; GO:0031047; GO:0031054; GO:0035197; GO:0035198; GO:0035280; GO:0042803; GO:0046782	Sp, F, H, K, L, Lu, M, S, T	ENSXETG00000012644	0.997025	999	0.022252093
Microcaecilia unicolor	***cyp17a1***	cytochrome P450 family 17 subfamily A member 1	GO:0004497; GO:0004508; GO:0005506; GO:0006694; GO:0007548; GO:0016491; GO:0016705; GO:0020037; GO:0042448; GO:0046872; GO:0047006; GO:0047442; GO:0055114;	K, T	ENSXETG00000015229	0.999271	42.68226	0.061514343

			GO:1903449					
Microcaecilia unicolor	***cybrd1***	cytochrome b reductase 1	GO:0000293; GO:0010039; GO:0016020; GO:0016021; GO:0031526; GO:0055114	F, H, K, L, Lu, T	ENSXETG00000018825	0.993126	999	0.024919723
Microcaecilia unicolor	***fyn***	FYN proto-oncogene Src family tyrosine kinase	GO:0000166; GO:0004672; GO:0004713; GO:0004715; GO:0005102; GO:0005524; GO:0006468; GO:0007169; GO:0016301; GO:0016310; GO:0016477; GO:0016740; GO:0030154; GO:0031234; GO:0038083; GO:0042127; GO:0045087	Sp, K, L, Lu, T	ENSXETG00000021344	0.998836	840.37469	1.65E-06
Microcaecilia unicolor	***srpk3***	SRSF protein kinase 3	GO:0000245; GO:0004672; GO:0004674; GO:0005524; GO:0005634; GO:0005737; GO:0006468; GO:0035556; GO:0050684	Sp, F, H, K, L, Lu, M, S, T	ENSXETG00000023173	0.997665	998.99805	1.62E-08
Microcaecilia unicolor	***stx3***	syntaxin 3	GO:0000149; GO:0005484; GO:0005622; GO:0005886; GO:0006886; GO:0006887; GO:0008021; GO:0016020; GO:0016021; GO:0016081; GO:0016192; GO:0031201; GO:0031629;	F, K, L, Lu, S, T	ENSXETG00000023730	0.995624	176.41269	0.002516965

			GO:0048278; GO:0061025; GO:0098793					
Microcaecilia unicolor	***plg***	uncharacteris-ed in Ensembl database; plasminogen (from NR database)	GO:0004252; GO:0006508; GO:0008233; GO:0008236; GO:0016787	L	ENSXETG00000033306	0.998386	46.69779	0.024919723
Microcaecilia dermatophaga	***tmprss2***	uncharacteris-ed in Ensembl database; transmembran e protease serine 2-like protein (from NR database)	GO:0004252; GO:0006508; GO:0008233; GO:0008236; GO:0016020; GO:0016021; GO:0016787	F, K, L, T	ENSXETG00000000063	0.991227	58.0989	0.093774823
Microcaecilia dermatophaga	***pacsin1***	uncharacteris-ed in Ensembl database; protein kinase C and casein kinase substrate in neurons 1-like protein (from NR database)	GO:0005768; GO:0030100	H, Lu, M, S, T	ENSXETG00000000295	0.998401	477.93978	0.094598672
Microcaecilia dermatophaga	***dnajc21***	DnaJ heat shock protein family	GO:0003676; GO:0008270; GO:0046872	Sp, F, H, K, L, Lu, M, S, T	ENSXETG00000000706	0.995016	82.97368	0.081176472
Microcaecilia dermatophaga	***mrc1***	mannose receptor C type 1	GO:0004888; GO:0005887; GO:0007165; GO:0016020; GO:0016021	F, K, L, Lu	ENSXETG00000001366	0.994313	31.14407	0.008226138
Microcaecilia dermatophaga	***hsdl2***	hydroxysteroi d dehydrogenas e like 2	-	F, K, L, Lu, M, S, T	ENSXETG00000002228	0.99907	149.55633	0.027606049
Microcaecilia dermatophaga	***mmp2***	matrix metallopeptida se 2	GO:0001945; GO:0004222; GO:0006508; GO:0008233; GO:0008237; GO:0008270; GO:0016787;	Sp, F, H, K, Lu, M, S, T	ENSXETG00000002801	1	56.88637	0.027606049

			GO:0031012; GO:0031290; GO:0046872					
Microcaecilia dermatophaga	***lcat***	lecithin-cholesterol acyltransferase	GO:0006629; GO:0008374	K, L, T	ENSXETG00000003085	0.999844	262.6812	0.063274157
Microcaecilia dermatophaga	***rock1***	Rho-associated coiled-coil containing protein kinase 1	GO:0000166; GO:0004672; GO:0004674; GO:0005524; GO:0005622; GO:0006468; GO:0007266; GO:0016301; GO:0016310; GO:0016740; GO:0017048; GO:0017049; GO:0030036; GO:0035556; GO:0046872; GO:0051492; GO:0051493; GO:2000114	Sp, F, H, K, L, Lu, S, T	ENSXETG00000003151	0.998392	19.86355	0.094598672
Microcaecilia dermatophaga	***tead4***	TEA domain family member 4	GO:0001085; GO:0003677; GO:0003700; GO:0005634; GO:0005667; GO:0006351; GO:0006355; GO:0035329; GO:0043565; GO:0044212; GO:0045944; GO:0048568	H, K, Lu, M, T	ENSXETG00000003395	0.99279	37.88286	0.013817142
Microcaecilia dermatophaga	***col12a1***	collagen type XII alpha 1	GO:0005615	K, L, Lu, M, S, T	ENSXETG00000003603	0.995985	16.58674	0.072180873
Microcaecilia dermatophaga	***cyp8b1***	cytochrome P450 family 8 subfamily B member 1	GO:0004497; GO:0005506; GO:0005783; GO:0005789; GO:0008397; GO:0016020; GO:0016021;	K, L, S	ENSXETG00000006173	0.99555	4.79268	0.027234823

			GO:0016491; GO:0016705; GO:0020037; GO:0046872; GO:0055114					
Microcaecilia dermatophaga	***adamts13***	ADAM metallopeptidase with thrombospondin type 1 motif 13	GO:0004222; GO:0005578; GO:0006508; GO:0008237; GO:0008270; GO:0031012; GO:0046872	F, K, L, Lu, M, T	ENSXETG00000006882	0.997414	60.36062	0.073264625
Microcaecilia dermatophaga	***inmt***	uncharacteris-ed in Ensembl database; indolethylamine N-methyltransferase (from NR database)	GO:0008168; GO:0032259	K, L, M, S	ENSXETG00000008551	0.988956	999	0.094598672
Microcaecilia dermatophaga	***pdgfd***	platelet derived growth factor D	GO:0005161; GO:0005615; GO:0007596; GO:0008083; GO:0008284; GO:0014068; GO:0016020; GO:0030335; GO:0031954; GO:0043406; GO:0048008; GO:0070374	F, H, K, Lu, M, S	ENSXETG00000010500	0.998821	999	0.098652291
Microcaecilia dermatophaga	***rad51ap1***	RAD51 associated protein 1	GO:0003690; GO:0003697; GO:0003723; GO:0005634; GO:0006281	F, K, L, Lu, M, S, T	ENSXETG00000011389	0.996857	998.99536	0.080010268
Microcaecilia dermatophaga	***asah1***	N-acylsphingosine amidohydrolase 1	GO:0005764; GO:0006629	Sp, F, K, L, Lu, S, T	ENSXETG00000012463	0.991958	10.4016	0.001107763
Microcaecilia dermatophaga	***tarbp2***	TAR RNA binding protein 2	GO:0003723; GO:0003725; GO:0005737; GO:0006417;	Sp, F, H, K, L, Lu, M, S, T	ENSXETG00000012644	0.997025	999	0.085852866

			GO:0016442; GO:0030422; GO:0030423; GO:0031047; GO:0031054; GO:0035197; GO:0035198; GO:0035280; GO:0042803; GO:0046782					
Microcaecilia dermatophaga	***lyz***	lysozyme	GO:0003796	Sp, F	ENSXETG00000013041	1	34.81264	0.025923145
Microcaecilia dermatophaga	***cfp***	complement factor properdin	-	Sp, H, K, L, Lu	ENSXETG00000013748	0.983925	999	0.092276399
Microcaecilia dermatophaga	***tm2d2***	TM2 domain containing 2	GO:0016020; GO:0016021	Sp, F, K, L, Lu, M, S, T	ENSXETG00000015155	0.999926	383.93701	0.094598672
Microcaecilia dermatophaga	***trip11***	thyroid hormone receptor interactor 11	GO:0005622	Sp, F, H, K, L, Lu, M, S, T	ENSXETG00000015833	0.998107	94.95153	0.032016564
Microcaecilia dermatophaga	***cers6***	ceramide synthase 6	GO:0003677; GO:0005634; GO:0005783; GO:0016020; GO:0016021; GO:0046513; GO:0050291	F, H, K, L, Lu, S, T	ENSXETG00000016207	0.993737	13.45155	0.072180873
Microcaecilia dermatophaga	***golga1***	golgin A1	GO:0005794	F, H, K, Lu, M, S, T	ENSXETG00000016840	0.997726	392.3178	0.009081857
Microcaecilia dermatophaga	***tspan9***	tetraspanin 9	GO:0005887; GO:0007166; GO:0016020; GO:0016021	Sp, F, H, K, Lu, M, S, T	ENSXETG00000016985	1	162.49378	0.080010268
Microcaecilia dermatophaga	***tcf7l2***	transcription factor 7-like 2	GO:0003677; GO:0005634; GO:0005667; GO:0006357; GO:0008013; GO:0016055; GO:0021986; GO:0035462; GO:0043565; GO:0044212; GO:0044333; GO:0060070;	Sp, F, K, L, Lu, S, T	ENSXETG00000018735	0.993536	999	1.13E-07

			GO:0060729; GO:2001237					
Microcaecilia dermatophaga	***rplp2***	ribosomal protein large P2	GO:0002181; GO:0003735; GO:0005622; GO:0005840; GO:0006414; GO:0022625; GO:0030529; GO:0043009	Sp, F, H, K, L, Lu, M, S, T	ENSXETG00000019024	0.989878	1.90298	0.027234823
Microcaecilia dermatophaga	***aldh1a1***	aldehyde dehydrogenase 1 family member A1	GO:0008152; GO:0016491; GO:0016620; GO:0018479; GO:0055114	K, L, T	ENSXETG00000019615	0.998999	999	0.027606049
Microcaecilia dermatophaga	***pfkp***	phosphofructokinase platelet	GO:0003872; GO:0005524; GO:0005737; GO:0006002; GO:0006096; GO:0061615	Sp, F, H, K, L, Lu, S, T	ENSXETG00000021922	0.997169	323.77006	4.59E-05
Microcaecilia dermatophaga	***fam3c***	family with sequence similarity 3 member C	GO:0005576; GO:0007275	Sp, F, H, K, L, Lu, S, T	ENSXETG00000022730	0.995627	108.57494	0.032016564
Microcaecilia dermatophaga	***folr1***	folate receptor 1	GO:0005542; GO:0008517; GO:0015884	F, H, K, L, Lu, M, S	ENSXETG00000023968	0.995358	63.04414	0.094598672
Microcaecilia dermatophaga	***sox17a***	SRY-box 17 alpha	GO:0003677; GO:0005634; GO:0006351; GO:0006355; GO:0007275; GO:0007369; GO:0007492; GO:0008013; GO:0016055; GO:0035469; GO:0043565; GO:0045893; GO:0045944; GO:0061371; GO:0070121	Sp, F, K, Lu, M, S	ENSXETG00000025005	0.991178	88.54354	1.37E-08
Microcaecilia dermatophaga	***nsl1***	MIS12 kinetochore complex	GO:0000070; GO:0000444	Sp, F, H, K, L, Lu, S, T	ENSXETG00000030886	0.999939	228.51919	0.027606049

		component						
Microcaecilia dermatophaga	***zcchc2***	zinc finger CCHC domain containing 2	GO:0003676; GO:0008270; GO:0035091	F, K, L, Lu, M, S, T	ENSXETG00000032980	0.997395	999	0.037285469
